# The impact of serum magnesium and calcium on the risk of epilepsy: A mendelian randomization study

**DOI:** 10.1111/cns.14248

**Published:** 2023-05-05

**Authors:** Xiaoming Guo, Yueli Zhu, Caidi Ying, Ke Xu, Yuan Hong

**Affiliations:** ^1^ Department of Neurosurgery The Second Affiliated Hospital, Zhejiang University School of Medicine Hangzhou China; ^2^ Department of Neurosurgery Tongde Hospital of Zhejiang Province Hangzhou China; ^3^ Department of Geriatrics The First Affiliated Hospital, Zhejiang University School of Medicine Hangzhou China

**Keywords:** epilepsy, seizure, serum calcium, serum magnesium

## Abstract

**Aims:**

To investigate the causal role of serum magnesium and calcium in epilepsy or any of its subtypes through Mendelian randomization (MR) approach.

**Methods:**

Single nucleotide polymorphisms (SNPs) associated with serum magnesium and calcium were used as the instrumental variables. MR analyses were performed using the summary‐level data for epilepsy extracted from International League Against Epilepsy Consortium (15,212 cases and 29,677 controls) to obtain the causal estimates. The analyses were replicated using FinnGen data (7224 epilepsy cases and 208,845 controls), and a meta‐analysis was then conducted.

**Results:**

The result of combined analyses showed that higher serum magnesium concentrations was associated with a reduced risk of overall epilepsy (odds ratios [OR] = 0.28, 95% confidence interval [CI], 0.12–0.62, *p* = 0.002). In ILAE, higher serum magnesium was suggestively associated with reduced risks of focal epilepsy (OR = 0.25, 95% CI 0.10–0.62, *p* = 0.003). However, the results cannot be repeated in sensitivity analyses. As for serum calcium, the results did not reach statistical significance with overall epilepsy (OR = 0.60, 95% CI, 0.31–1.17, *p* = 0.134). However, genetically predicted serum calcium concentrations showed an inverse association with risk of generalized epilepsy (OR = 0.35, 95% CI, 0.17–0.74, *p* = 0.006).

**Conclusion:**

The current MR analysis did not support a causal relationship between serum magnesium and epilepsy, but showed a causally negative association between genetically determined serum calcium and generalized epilepsy.

## 1 INTRODUCTION

Epilepsy is a heterogeneous and complex brain condition with a genetic predisposition, which is associated with various risk factors.^1^ The etiology for epilepsy is multifactorial, including genetic causation, cerebrovascular disorders, cerebral trauma, cerebral infections, neurodegenerative diseases, and so on.^2^ Despite the rapid progress in investigatory technology, the etiology and underlying pathology remained undiscovered in approximately 50% of newly diagnosed epilepsy cases.^3^ Based on the Global Burden of Disease Study 2010, epilepsy was ranked as the second most burdensome neurological diseases worldwide as far as disability‐adjusted life years were concerned.^4^ Besides, 80% of epileptics live in low‐ and middle‐income countries where treatment is probably not well used.^5^ Consequently, it is of great value to evaluate potential risk factors for epilepsy, which would substantially reduce the incidence of morbidity and disability.

Magnesium and calcium are two vital minerals that have been reported to participate in the pathogenetic processes of neurological disorders, such as ischemic stroke, migraine, and depression.^6^, ^7^, ^8^ Previous observational studies have revealed that decreased concentrations of serum magnesium were associated with an increased risk of epilepsy.^9^, ^10^, ^11^ A 22‐year follow‐up study also revealed that higher oral magnesium supplementation was related to a lower risk of adult epilepsy.^12^ Recently, animal research showed a certain relationship between magnesium homeostasis and acute or chronic seizures.^13^ Likewise, lower serum calcium concentrations was reported to be associated with a higher risk of epilepsy.^9^, ^10^, ^14^ As we know, hypomagnesaemia can cause serum calcium levels to decline by affecting the synthesis or secretion of parathyroid hormone. In addition, low levels of serum calcium, together with low magnesium levels, can lead to membrane hyperexcitability in neurons, which has been found to be strongly related to seizures in both children and adults.^9^ Besides, the decrease in serum calcium levels in epileptics may be attributed to the rapid inflow of calcium ions from blood into tissue.^9^ However, a previous meta‐analysis including 13 studies showed no significant difference in serum magnesium and calcium between epileptics and controls.^15^ Therefore, there is no sufficient evidence regarding whether serum magnesium and calcium can be causally involved in the emergence and development of epilepsy and its subtypes. While in clinical practice, magnesium and calcium, as primary preventions along with therapeutic intervention for epilepsy, has not been attached enough importance especially in adults.

Mendelian randomization (MR) is a genetic epidemiologic method by using genetic variants associated with exposures, which can avoid many of the potential methodological limitations of observational studies, such as reverse causation bias and confounding.^16^ We conducted this MR study to examine the causal relationship between epilepsy and serum magnesium and calcium concentrations.

## 2 METHODS

### 2.1 Genetic instruments selection and data sources

The MR study builds on three predominant assumptions (Figure S1). We obtained summary‐level data for all single nucleotide polymorphisms (SNPs) associated with serum magnesium and calcium in genome‐wide association studies (GWAS), based on 23,829 and 305,349 individuals of European ancestry, respectively.^17^, ^18^ Six independent SNPs associated with serum magnesium concentrations and two SNPs which were implicated as magnesium transporters were proposed as instrumental variables.^18^ Two SNPs were associated with serum magnesium concentrations after applying a Bonferroni correction for the number of genetic variants examined in each region. Similarly, 142 SNPs associated with serum calcium concentrations were used as instrumental variables.^18^ The variants of serum calcium explained 5.8% variance. Moreover, these SNPs associated with serum magnesium and calcium were clumped for independence (*r* ^*2*^ < 0.1; region size, 3000 kb) according to the Europeans data from the 1000 Genomes Project. If these included SNPs were not available in the outcome datasets, proxy SNPs (*r* ^*2*^ > 0.8) were acquired online as replacements (ldlink.nci.nih.gov/).

Genetic results for epilepsy were examined in ILEA, a collaboration of the International League Against Epilepsy, which brought together genome‐wide data on a total of 15,212 epilepsy cases and 29,677 controls of about 86% European ancestry.^19^ As for epilepsy subtypes, summary statistics for focal epilepsy (9671 cases) and generalized epilepsy (3769 cases) were also obtained.^19^ More detailed information including data collection, study design and genetic data quality control were available in the original works. The effect size and standard error for each SNP were estimated using the equation described by Zhu et al.^20^ To further verify the reliability of the results, summary statistics for all epilepsy were extracted from the publicly available data through FinnGen Data Freeze 6 (7224 cases and 208,845 controls).^21^

### 2.2 Statistical analysis

In the MR analyses, we applied the random‐effects inverse‐variance weighted (IVW) approach to obtain causal estimates. We conducted several sensitivity analyses to identify potential pleiotropy: (1) Cochran's *Q* test, which was used to evaluate the heterogeneity among different instrumental variables^22^; (2) weighted median method, which allowed less than 50% of the genetic variants to be invalid instrumental variables^23^; (3) MR‐Egger method, which can detect and adjust pleiotropic bias.^24^ To further control potential pleiotropy, we used the MR Pleiotropy Residual Sum and Outlier (MR‐PRESSO) method to conduct a global test of heterogeneity and identify horizontal pleiotropy.^25^ Once the pleiotropic outlier instruments were identified, a repeated IVW analysis after removing these outlier instruments would be performed. To increase the robustness of the results, analyses were also performed using summary‐level data from FinnGen and a fixed‐effects meta‐analysis was conducted to combine results from ILAE and FinnGen consortium.

Odds ratios (ORs) were presented for each 1 SD difference in serum magnesium concentrations (equating to about 0.1 mmol/L) and serum calcium concentrations (equating to about 0.5 mg/dL). All tests were two sided and the Bonferroni‐corrected significance threshold was set to *p* < 0.05/8 (correcting for two exposures and four outcomes). The *p* values between 0.05/8 and 0.05 were defined as suggestive associations between exposures and outcomes. We perform power calculation for this MR analyses using an online tool (https://sb452.shinyapps.io/power). All analyses were conducted by using TwoSampleMR and MR‐PRESSO packages in R software (Version 4.1.3).^25^, ^26^

## 3 RESULTS

The characteristics of SNPs on serum magnesium and calcium were shown in Table S1. Among the SNPs associated with serum magnesium and calcium concentration, SNPs unavailable in the outcome in the datasets and proxy genetic variants in linkage disequilibrium (*r* ^*2*^ > 0.8) was shown in Tables S2–S4.

### 3.1 Serum magnesium and epilepsy

**FIGURE 1:**
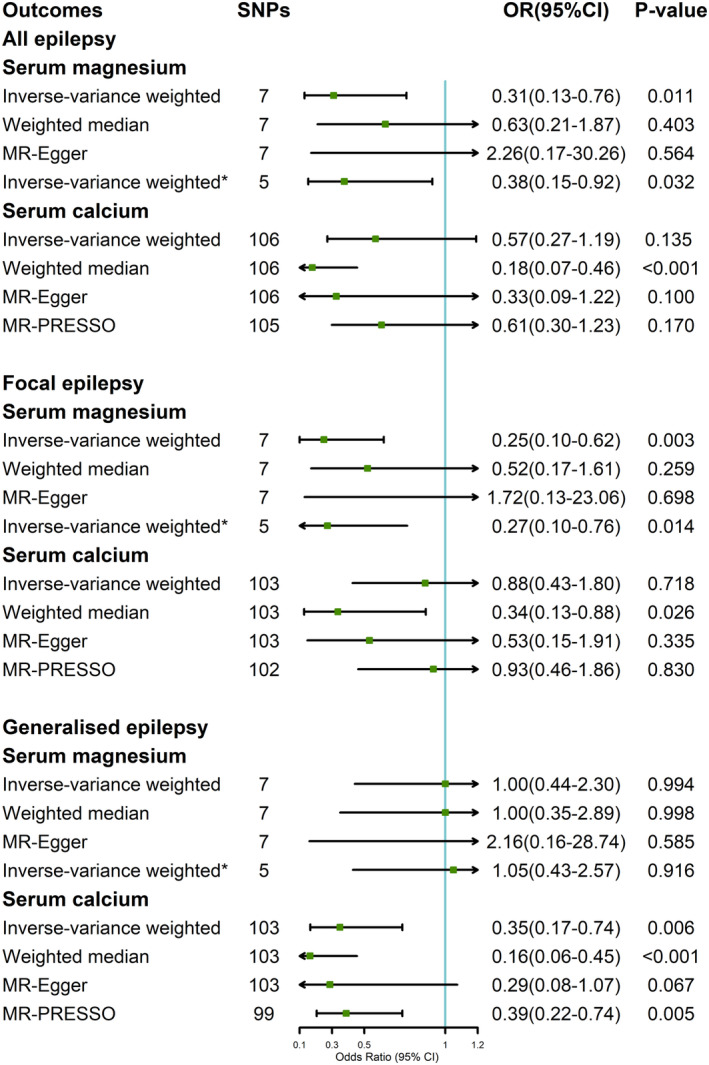
Association between genetically predicted serum magnesium and calcium concentrations and epilepsy and its subtypes in ILAE. *Inverse‐variance weighted excluding two genetic variants (rs3740393 and rs994430) in or near known magnesium transport genes and associated with serum magnesium after applying a Bonferroni correction for the number of genetic variants assessed in each region. CI, confidence interval; OR, odds ratio; SNP, single nucleotide polymorphism.

In the random‐effects IVW estimates, suggestive evidence was found between genetically determined increased serum magnesium concentrations and a reduced risk of overall epilepsy (Figure 1), with an OR for each 0.1 mmol/L increment of 0.31 (95% confidence interval [CI] 0.13–0.76, *p* = 0.011). However, similar relationship was not found in weighted median and MR‐Egger estimates. There was no evidence of horizontal pleiotropy (*p* for intercept = 0.176) or heterogeneity estimated by the Cochran's *Q* test (*p* for Cochran's *Q* = 0.337). Among the seven SNPs, no significant potential outlier was found in the MR‐PRESSO test.

In the subgroup analysis of focal epilepsy, the OR per 0.1 mmol/L (about 1 SD) difference in serum magnesium concentrations was 0.25 (95% CI 0.10–0.62, *p* = 0.003) in the IVW estimates. However, in weighted median and MR‐Egger estimates, such causal association was also not found. There was no evidence of horizontal pleiotropy (P for intercept = 0.182) or heterogeneity estimated by the Cochran's *Q* test (P for Cochran's *Q* = 0.299).

### 3.2 Serum calcium and epilepsy

This MR study suggested that genetically predicted serum calcium was not causally associated with epilepsy or subtype of focal epilepsy based on the IVW method (Figure 1). However, an inverse association was observed between genetically determined serum calcium and generalized epilepsy in the random‐effects IVW estimates (OR = 0.35, 95CI% 0.17–0.74, *p* = 0.006). Similar results were observed using the weighted median (OR = 0.16, 95% CI, 0.06–0.45; *p* < 0.001), MR‐Egger methods (OR = 0.29, 95% CI, 0.08–1.07; *p* = 0.067) and MR‐PRESSO (OR = 0.39, 95CI% 0.22–0.74, *p* = 0.005). There was no evidence of horizontal pleiotropy (P for intercept = 0.724). The Cochran Q test indicated significant heterogeneity (P for Cochran's *Q* < 0.001). Several outliers were excluded in the MR‐PRESSO test and were shown in Table S4.

### 3.3 Replication and meta‐analyses

**FIGURE 2:**
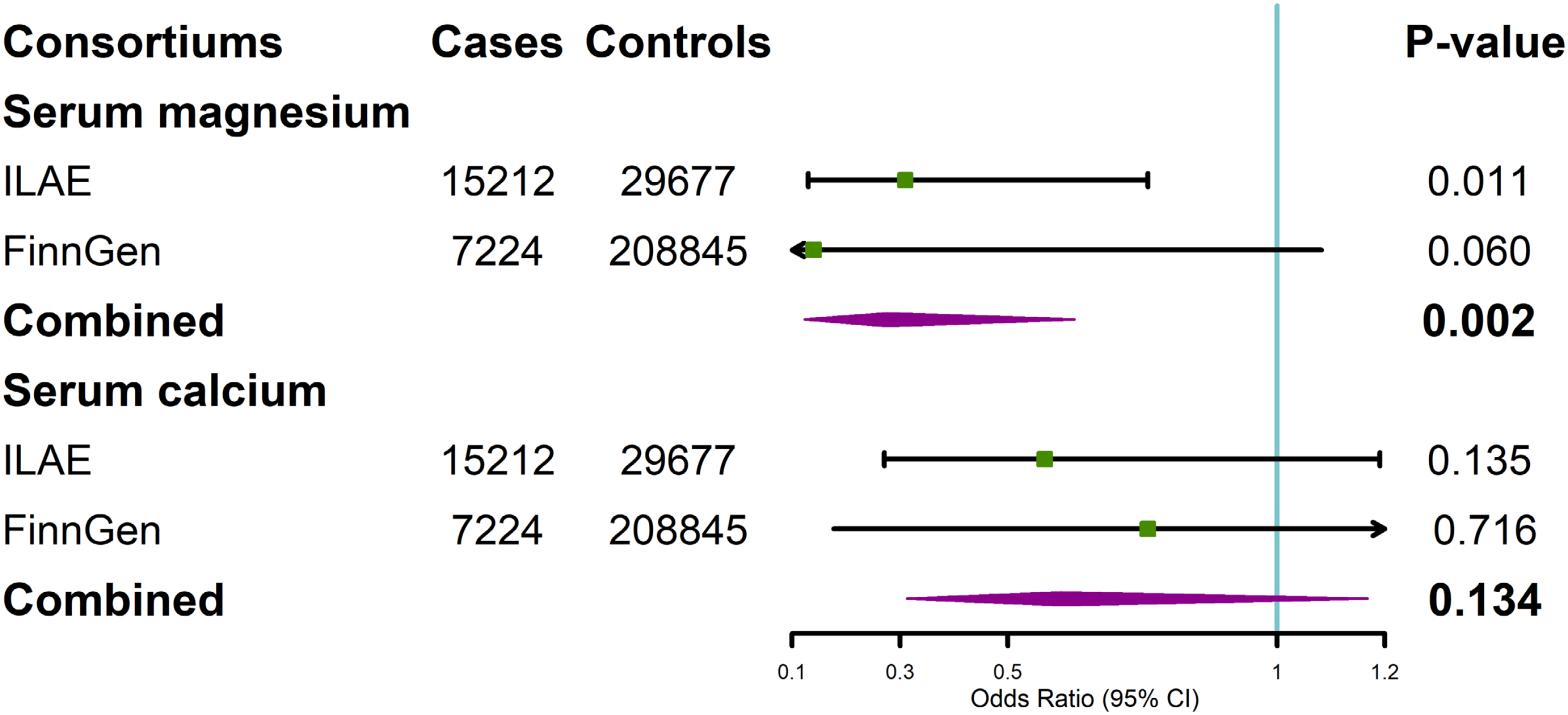
Association between genetically predicted serum magnesium and calcium concentrations and epilepsy in ILAE, FinnGen, and combined analyses of both samples. ILAE, International League Against Epilepsy.

In the replicated analyses of summary statistics from FinnGen, the results yielded the same direction of effects estimate, but did not reach statistical significance (Figure S2). The combined associations of genetically predicted serum magnesium concentrations with overall epilepsy reached at *p* = 0.002 (OR = 0.28, 95% CI, 0.12–0.62) in meta‐analyses of data from two consortiums (Figure 2). As for genetic predicted serum calcium concentrations, the combined result also confirmed the lack of causality (OR = 0.60, 95% CI, 0.31–1.17, *p* = 0.134).

## 4 DISCUSSION

We conducted this MR study to test for causal association of serum magnesium and calcium in relation to epilepsy and its subtypes. Results from IVW and combined analyses revealed suggestive association between genetically determined serum magnesium and epilepsy, but the results cannot be repeated in sensitivity analyses. Meanwhile, this MR study also provided evidence that serum calcium was casually associated with generalized epilepsy.

Magnesium is the second most common intracellular cation after potassium as well as the fourth most abundant mineral in the human body. Magnesium plays the essential role in enzyme systems and transport of potassium and calcium ion within the nervous system.^27^, ^28^ Animal research suggested that magnesium could potentially modulate seizure activity due to its function to antagonize the excitatory calcium influx under the N‐methyl‐d‐aspartate receptor.^29^ Magnesium deficiency lowered the thresholds and latencies of seizure in rats, which can increase after three‐week oral magnesium supplementation.^30^ Further studies showed that oral magnesium organic salts can inhibit N‐methyl‐D‐aspartate induced convulsions in mice in a dose‐dependent manner, which was similar to the N‐methyl‐D‐aspartate receptor blocker, MK‐801.^31^ Observational studies have investigated the association between serum magnesium and epilepsy. Despite of significant results, the casual relationship still could not be drawn.^9^, ^10^, ^11^ In a randomized open‐label study, intravenous magnesium supplementation, as an additional treatment together with adrenocorticotropic hormone, improved the seizure‐free rate at 4–28 weeks seizures compared with adrenocorticotropic hormone monotherapy among children with infantile.^32^ A retrospective study showed adjunctive magnesium supplementation could reduce the seizure days per month for patients with drug‐resistant seizures.^33^ Although human studies and animal models research have provided a theoretical basis for the therapeutic and prophylactic benefits of magnesium in treating epilepsy, there were still no randomized and controlled trials proposed to evaluate this hypothesis. Using two‐sample MR analyses, our results suggested a potential negative relationship between serum magnesium and overall epilepsy and focal epilepsy, despite of the absence of significance in the sensitivity analyses. Then, we pooled another large dataset (FinnGen) to further confirm the potential link, which revealed a similar phenomenon. However, we supposed that caution should be taken when extrapolating these findings to the clinical situation.

Calcium is also an essential element in human body. Calcium can regulate neuronal excitability by altering γ‐aminobutyric acid type A receptors recycling in the epileptic condition.^34^ In a cohort of Black Africans, low serum calcium concentrations were associated with idiopathic generalized epileptic, compared with symptomatic epileptic seizures and healthy controls.^10^ In another cohort of 200 patients with genetic generalized epilepsy, similar association was detected (1.85 ± 0.33 mmol/L vs. 2.27 ± 0.22 mmol/L).^9^ However, decreased levels of serum calcium was not observed in a few studies.^35^, ^36^ One possibility is that most of these included patients in the studies were focal epilepsy, while the decrease of serum calcium levels might only be observed in patients with generalized epilepsy. Although previous literatures reported that antiepileptic drugs altered calcium metabolism in patients with epilepsy, the alternation cannot directly influence the levels of serum calcium.^36^, ^37^, ^38^ This MR analyses verified the association between serum calcium concentrations and risk of generalized epilepsy.

The strength of this study was that the use of two datasets to assess the robustness of our results. However, there were several limitations in our study. First, the results of serum magnesium were not significant in the sensitivity analyses. One possible reason was analyses of instruments for a number of serum magnesium may be affected by heterogeneity and horizontal pleiotropy. Moreover, the included SNPs only explained a small part of the variance (1.6%) in serum magnesium levels, although the power for MR analysis is largely influenced by the strength of SNP‐outcome association.^39^ Further analyses with larger GWAS are needed to illustrate the mechanisms between serum magnesium and epilepsy. Second, serum magnesium and calcium may not be good indicators for the total body content of magnesium and calcium because they are not sensitive to show intracellular magnesium and calcium pooling. Third, most of participants in this study were of European descent, which limited our findings to extend to other ancestries. Finally, potential pleiotropic SNPs might affect our results. Thus, we used weighted median and MR‐Egger approaches to minimize the possible violations of the MR assumptions. Moreover, other methods were also applied to identify potential pleiotropy of SNPs.

In conclusion, current MR analysis did not support a causal relationship between serum magnesium and epilepsy. In contrast, serum calcium was causally associated with a reduced risk of generalized epilepsy. Further replication of these analyses using even larger GWASs are required.

## AUTHOR CONTRIBUTIONS

Xiaoming Guo: Drafting/revision of the manuscript, study design, and analysis and interpretation of data. Yueli Zhu: Drafting/revision of the manuscript and analysis and interpretation of data. Caidi Ying: Acquisition of data. Ke Xu: Acquisition of data. Yuan Hong: Revision of the manuscript.

## FUNDING INFORMATION

This study was supported by the National Natural Science Foundation of China (81870964).

## CONFLICT OF INTEREST STATEMENT

None of the authors has any conflict of interest to disclose.

## Supporting information


Appendix S1

## Data Availability

Genetic variants used can be obtained in the original studies (doi.org/10.1371/journal.pgen.1001045, doi.org/10.1161/CIRCGEN.120.003231). The summary statistics from the GWAS studies for epilepsy are publicly available through the ILEA (www.epigad.org/gwas_ilae2018_16loci.html) and FinnGen (www.finngen.fi).
