# Supplementary material for: The impact of serum magnesium and calcium on the risk of epilepsy: A mendelian randomization study
Source: CNS Neurosci Ther. 2023 May 5;29(10):3062–7. doi: 10.1111/cns.14248 (PMC10493656; doi:10.1111/cns.14248)
Supplement: Supplementary file 1 — Appendix S1 [file CNS-29-3062-s001.docx]

eTable 1. Studies and datasets adopted in the MR analyses.

| Outcomes | Data source | Sample size(cases/controls) | Sample overlap | Ancestry |
| --- | --- | --- | --- | --- |
| Epilepsy | ILAE consortium | 15,212 cases/29,677 controls | 0 | ~86% Europeans |
| Epilepsy | FinnGen consortium R6 release | 4,588 cases/144,780 controls | 0 | Europeans |
| Serum magnesium | CHARGE and replication studies | 23,829 | 0 | Europeans |
| Serum calcium | UK Biobank | 305,349 | 0 | Europeans |

eTable 2. Characteristics of the single nucleotide polymorphisms used as instrumental variables for serum magnesium concentrations and their associations with epilepsy and its subtypes in ILAE.

|  |  |  |  | Serum magnesium concentrations | |  | Epilepsy (focal and generalized) | |
| --- | --- | --- | --- | --- | --- | --- | --- | --- |
| SNP | Chr | Gene | EA | β (SE) | P value |  | β (SE) | P value |
| rs4072037 | 1 | *MUC1* | C | -0.010 (0.001) | 2.01E-36 |  | 0.003 (0.007) | 0.647 |
| rs13146355 | 4 | *SHROOM3* | G | -0.005 (0.001) | 6.27E-13 |  | 0.002 (0.007) | 0.759 |
| rs3925584 | 11 | *DCDC5* | C | -0.006 (0.001) | 5.20E-16 |  | 0.010 (0.007) | 0.136 |
| rs7965584^a^ | 12 | *ATP2B1* | G | -0.007 (0.001) | 1.05E-16 |  | 0.016 (0.008) | 0.035 |
| rs448378 | 3 | *MDS1* | G | -0.004 (0.001) | 1.25E-08 |  | 0.006 (0.007) | 0.401 |
| rs3740393^b^ | 10 | *CNNM2* | G | -0.006 (0.001) | 8.58E-07 |  | 0.003 (0.009) | 0.714 |
| rs994430^b^ | 2 | *CNNM3* | A | -0.004 (0.001) | 1.53E-04 |  | 0.017 (0.007) | 0.011 |

| Focal Epilepsy | |  | Generalized Epilepsy | |
| --- | --- | --- | --- | --- |
| β (SE) | P value |  | β (SE) | P value |
| 0.005 (0.007) | 0.431 |  | 6.69E-06 (0.007) | 0.999 |
| -4.72E-05 (0.007) | 0.994 |  | 0.005 (0.007) | 0.428 |
| 0.014 (0.007) | 0.038 |  | 0.0003 (0.007) | 0.962 |
| 0.018 (0.008) | 0.017 |  | -0.005 (0.008) | 0.528 |
| 0.011 (0.007) | 0.090 |  | -0.003 (0.007) | 0.640 |
| 0.004 (0.009) | 0.655 |  | -0.008 (0.009) | 0.385 |
| 0.014 (0.007) | 0.035 |  | 0.009 (0.009) | 0.194 |

Abbreviations: Chr = Chromosome, EA = effect allele; SE = standard error.

^a^The single nucleotide polymorphism was unavailable in the ILAE datasets but a proxy genetic variant ( rs11105429) in linkage disequilibrium was identified and replaced this genetic variant.

^b^Used in complementary analyses that included genetic variants in or near known magnesium transport genes and associated with serum magnesium after applying a Bonferroni correction for the number of genetic variants assessed in each region.

eTable 3. Characteristics of the single nucleotide polymorphisms used as instrumental variables for serum magnesium concentrations and their associations with epilepsy in FinnGen.

|  |  |  |  | Serum magnesium concentrations | |  | Epilepsy | |
| --- | --- | --- | --- | --- | --- | --- | --- | --- |
| SNP | Chr | Gene | EA | β (SE) | P value |  | β (SE) | P value |
| rs4072037 | 1 | *MUC1* | C | -0.010 (0.001) | 2.01E-36 |  | 0.029 (0.017) | 0.093 |
| rs13146355 | 4 | *SHROOM3* | G | -0.005 (0.001) | 6.27E-13 |  | -0.013 (0.017) | 0.454 |
| rs11144134 | 9 | *TRPM6* | T | -0.011 (0.001) | 8.21E-15 |  | -0.025 (0.035) | 0.485 |
| rs3925584 | 11 | *DCDC5* | C | -0.006 (0.001) | 5.20E-16 |  | 0.019 (0.017) | 0.285 |
| rs7965584^a^ | 12 | *ATP2B1* | G | -0.007 (0.001) | 1.05E-16 |  | 0.011 (0.018) | 0.554 |
| rs448378 | 3 | *MDS1* | G | -0.004 (0.001) | 1.25E-08 |  | 0.006 (0.007) | 0.401 |
| rs3740393 | 10 | *CNNM2* | G | -0.006 (0.001) | 8.58E-07 |  | 0.003 (0.009) | 0.714 |
| rs6746896 | 2 | *CNNM4* | A | -0.004 (0.001) | 7.01E-05 |  | 0.017 (0.007) | 0.011 |

Abbreviations: Chr = Chromosome, EA = effect allele; SE = standard error.

^a^The single nucleotide polymorphism was unavailable in the FinnGen datasets but a proxy genetic variant (rs11105429) in

linkage disequilibrium was identified and replaced this genetic variant.

eTable 4. Characteristics of the single nucleotide polymorphisms used as instrumental variables for serum calcium concentrations and their associations with epilepsy in ILAE and FinnGen.

| SNP | Chr | BETA_epilepsy | SE_epilepsy | BETA_focal  _epilepsy | SE_focal_  epilepsy | BETA_genera-  lized_epilepsy | SE_generalized  _epilepsy | BETA_FinnGen | SE_FinnGen | Proxy_ILEA | Proxy_  FinnGen |
| --- | --- | --- | --- | --- | --- | --- | --- | --- | --- | --- | --- |
| rs61766352 | 1 | -0.00238 | 0.000257 | -0.00238 | 0.000257 | -0.00238 | 0.000257 | -0.00238 | 0.000257 |  |  |
| rs12132412 | 1 | 0.002386 | 0.000234 | 0.002386 | 0.000234 | 0.002386 | 0.000234 | 0.001839 | 0.000229 |  |  |
| rs111745074 | 1 | -0.00155 | 0.000278 | -0.00155 | 0.000278 | -0.00155 | 0.000278 | 0.002386 | 0.000234 | rs66531516 | rs66531516 |
| rs112174050 | 1 | 0.009962 | 0.000733 | 0.009962 | 0.000733 | 0.009962 | 0.000733 | -0.00155 | 0.000278 |  |  |
| rs148756772 | 1 | 0.006967 | 0.000655 | 0.006967 | 0.000655 | 0.006967 | 0.000655 | 0.002805 | 0.000231 |  |  |
| rs760077 | 1 | -0.00251 | 0.000231 | -0.00251 | 0.000231 | -0.00251 | 0.000231 | 0.009962 | 0.000733 | rs6676150 | |
| rs3011 | 1 | -0.0019 | 0.000235 | -0.0019 | 0.000235 | -0.0019 | 0.000235 | 0.006967 | 0.000655 | rs1891562 | rs1891562 |
| rs1260326 | 2 | -0.00445 | 0.000231 | -0.00445 | 0.000231 | -0.00445 | 0.000231 | -0.00148 | 0.00025 |  |  |
| rs111712978 | 2 | -0.0042 | 0.000637 | -0.0042 | 0.000637 | -0.0042 | 0.000637 | 0.00158 | 0.000272 |  |  |
| rs1448208 | 2 | -0.00139 | 0.000242 | -0.00139 | 0.000242 | -0.00139 | 0.000242 | 0.001402 | 0.000231 |  |  |
| rs11673819 | 2 | -0.00219 | 0.000249 | -0.00219 | 0.000249 | -0.00219 | 0.000249 | -0.00251 | 0.000231 | rs3889224 | |
| rs13322435 | 3 | 0.001779 | 0.000231 | 0.001779 | 0.000231 | 0.001779 | 0.000231 | 0.002059 | 0.000254 |  |  |
| rs2593813 | 3 | 0.003279 | 0.000234 | 0.003279 | 0.000234 | 0.003279 | 0.000234 | 0.001539 | 0.000231 |  |  |
| rs13108218 | 4 | -0.00349 | 0.000235 | -0.00349 | 0.000235 | -0.00349 | 0.000235 | 0.001792 | 0.000235 |  |  |
| rs6841258 | 4 | -0.00369 | 0.000303 | -0.00369 | 0.000303 | -0.00369 | 0.000303 | -0.0019 | 0.000235 |  |  |
| rs4864709 | 4 | 0.001293 | 0.000235 | 0.001293 | 0.000235 | 0.001293 | 0.000235 | -0.0016 | 0.000227 |  |  |
| rs13111128 | 4 | -0.0013 | 0.000249 | -0.0013 | 0.000249 | -0.0013 | 0.000249 | -0.00445 | 0.000231 | rs34534635 | |
| rs13107325 | 4 | -0.00623 | 0.000428 | -0.00623 | 0.000428 | -0.00623 | 0.000428 | 0.001244 | 0.000227 |  |  |
| rs6556313 | 5 | 0.001649 | 0.000241 | 0.001649 | 0.000241 | 0.001649 | 0.000241 | 0.001816 | 0.00031 | rs4976688 | |
| rs2327774 | 6 | -0.00182 | 0.000238 | -0.00182 | 0.000238 | -0.00182 | 0.000238 | 0.002016 | 0.000316 |  |  |
| rs5745687 | 7 | 0.002584 | 0.000457 | 0.002584 | 0.000457 | 0.002584 | 0.000457 | 0.001388 | 0.000246 |  |  |
| rs511220 | 7 | 0.001341 | 0.000242 | 0.001341 | 0.000242 | 0.001341 | 0.000242 | -0.0042 | 0.000637 |  |  |
| rs34372369 | 7 | 0.003306 | 0.000512 | 0.003306 | 0.000512 | 0.003306 | 0.000512 | -0.0035 | 0.000232 |  |  |
| rs62493995 | 8 | -0.00193 | 0.000259 | -0.00193 | 0.000259 | -0.00193 | 0.000259 | -0.00139 | 0.000242 |  |  |
| rs9657190 | 8 | 0.001851 | 0.000263 | 0.001851 | 0.000263 | 0.001851 | 0.000263 | 0.002143 | 0.000228 | rs2411256 | |
| rs74677734 | 8 | 0.003259 | 0.000526 | 0.003259 | 0.000526 | 0.003259 | 0.000526 | -0.00219 | 0.000249 |  |  |
| rs6983263 | 8 | -0.00132 | 0.000229 | -0.00132 | 0.000229 | -0.00132 | 0.000229 | -0.00139 | 0.000231 | rs6994620 | |
| rs3133528 | 8 | 0.001792 | 0.000334 | 0.001792 | 0.000334 | 0.001792 | 0.000334 | 0.001483 | 0.000228 | rs11785473 | rs11785473 |
| rs6993770 | 8 | -0.00188 | 0.00025 | -0.00188 | 0.00025 | -0.00188 | 0.00025 | 0.002221 | 0.000337 | rs2343592 | |
| rs62522556 | 8 | 0.001577 | 0.000232 | 0.001577 | 0.000232 | 0.001577 | 0.000232 | -0.00375 | 0.000229 | rs11136336 | rs11136336 |
| rs77798356 | 9 | 0.00211 | 0.000357 | 0.00211 | 0.000357 | 0.00211 | 0.000357 | 0.002422 | 0.000439 |  |  |
| rs148349564 | 9 | -0.00338 | 0.00042 | -0.00338 | 0.00042 | -0.00338 | 0.00042 | -0.00218 | 0.000236 |  |  |
| rs4744854 | 9 | -0.00273 | 0.000235 | -0.00273 | 0.000235 | -0.00273 | 0.000235 | -0.00191 | 0.000268 |  |  |
| rs13283282 | 9 | -0.00199 | 0.000321 | -0.00199 | 0.000321 | -0.00199 | 0.000321 | -0.00332 | 0.000347 |  |  |
| rs9411378 | 9 | -0.00162 | 0.000284 | -0.00162 | 0.000284 | -0.00162 | 0.000284 | 0.001386 | 0.000227 | rs550057 | rs550057 |
| rs36225067 | 11 | 0.005728 | 0.000722 | 0.005728 | 0.000722 | 0.005728 | 0.000722 | -0.0017 | 0.000234 |  |  |
| rs7115200 | 11 | -0.00146 | 0.000229 | -0.00146 | 0.000229 | -0.00146 | 0.000229 | 0.004122 | 0.00043 |  |  |
| rs561830 | 11 | -0.00216 | 0.000262 | -0.00216 | 0.000262 | -0.00216 | 0.000262 | 0.001836 | 0.000229 | rs1047417 | |
| rs7928577 | 11 | -0.00549 | 0.000433 | -0.00549 | 0.000433 | -0.00549 | 0.000433 | -0.01767 | 0.000335 |  |  |
| rs117213754 | 12 | 0.00825 | 0.000941 | 0.00825 | 0.000941 | 0.00825 | 0.000941 | 0.002542 | 0.000256 |  |  |
| rs4763297 | 12 | 0.001603 | 0.00023 | 0.001603 | 0.00023 | 0.001603 | 0.00023 | -0.00128 | 0.000227 |  |  |
| rs12369443 | 12 | -0.00164 | 0.000286 | -0.00164 | 0.000286 | -0.00164 | 0.000286 | 0.001779 | 0.000231 |  |  |
| rs12320328 | 12 | -0.00265 | 0.000408 | -0.00265 | 0.000408 | -0.00265 | 0.000408 | 0.003279 | 0.000234 |  |  |
| rs79295634 | 12 | 0.002446 | 0.000451 | 0.002446 | 0.000451 | 0.002446 | 0.000451 | -0.00349 | 0.000235 | rs77777887 | |
| rs17884869 | 12 | -0.00958 | 0.00073 | -0.00958 | 0.00073 | -0.00958 | 0.00073 | 0.001386 | 0.000235 |  |  |
| rs78844280 | 13 | -0.00432 | 0.000771 | -0.00432 | 0.000771 | -0.00432 | 0.000771 | -0.00369 | 0.000303 |  |  |
| rs11625026 | 14 | 0.001541 | 0.000244 | 0.001541 | 0.000244 | 0.001541 | 0.000244 | 0.001293 | 0.000235 |  |  |
| rs28929474 | 14 | 0.01164 | 0.000817 | 0.01164 | 0.000817 | 0.01164 | 0.000817 | -0.0013 | 0.000249 |  |  |
| rs75153054 | 14 | -0.00183 | 0.000271 | -0.00183 | 0.000271 | -0.00183 | 0.000271 | -0.00623 | 0.000428 |  |  |
| rs55754498 | 15 | -0.00301 | 0.000507 | -0.00301 | 0.000507 | -0.00301 | 0.000507 | -0.00165 | 0.000267 |  |  |
| rs12148513 | 15 | 0.001276 | 0.000236 | 0.001276 | 0.000236 | 0.001276 | 0.000236 | -0.00131 | 0.00023 |  |  |
| rs41278174 | 16 | 0.004561 | 0.000695 | 0.004561 | 0.000695 | 0.004561 | 0.000695 | 0.00452 | 0.000591 |  |  |
| rs12922549 | 16 | -0.00225 | 0.000272 | -0.00225 | 0.000272 | -0.00225 | 0.000272 | 0.001531 | 0.000229 | rs28621883 | |
| rs34422500 | 16 | 0.002444 | 0.000347 | 0.002444 | 0.000347 | 0.002444 | 0.000347 | -0.00133 | 0.000227 |  |  |
| rs11078597 | 17 | 0.004854 | 0.000291 | 0.004854 | 0.000291 | 0.004854 | 0.000291 | 0.001483 | 0.000261 | rs9912287 | |
| rs5033 | 17 | -0.00161 | 0.00025 | -0.00161 | 0.00025 | -0.00161 | 0.00025 | -0.00231 | 0.000255 | rs10208 | rs10208 |
| rs77542162 | 17 | -0.0086 | 0.000762 | -0.0086 | 0.000762 | -0.0086 | 0.000762 | 0.001649 | 0.000241 |  |  |
| rs689049 | 18 | 0.001359 | 0.000232 | 0.001359 | 0.000232 | 0.001359 | 0.000232 | 0.003858 | 0.000298 | rs11152156 | |
| rs190424317 | 19 | -0.00569 | 0.000369 | -0.00569 | 0.000369 | -0.00569 | 0.000369 | -0.00133 | 0.000243 |  |  |
| rs7252372 | 19 | 0.001276 | 0.000232 | 0.001276 | 0.000232 | 0.001276 | 0.000232 | -0.00346 | 0.000227 |  |  |
| rs143333049 | 19 | 0.004958 | 0.000595 | 0.004958 | 0.000595 | 0.004958 | 0.000595 | 0.001687 | 0.000232 |  |  |
| rs142888784 | 19 | -0.00876 | 0.000841 | -0.00876 | 0.000841 | -0.00876 | 0.000841 | -0.00209 | 0.000245 |  |  |
| rs73036517 | 19 | -0.00146 | 0.00026 | -0.00146 | 0.00026 | -0.00146 | 0.00026 | -0.00205 | 0.000245 |  |  |
| rs12983362 | 19 | 0.002122 | 0.000263 | 0.002122 | 0.000263 | 0.002122 | 0.000263 | -0.00253 | 0.000232 |  |  |
| rs73078112 | 20 | 0.004687 | 0.0007 | 0.004687 | 0.0007 | 0.004687 | 0.0007 | -0.00182 | 0.000238 |  |  |
| rs3091842 | 20 | 0.008099 | 0.00057 | 0.008099 | 0.00057 | 0.008099 | 0.00057 | 0.001881 | 0.000249 |  |  |
| rs3787267 | 20 | -0.00136 | 0.000241 | -0.00136 | 0.000241 | -0.00136 | 0.000241 | 0.002856 | 0.000228 | rs6066153 | |
| rs185799410 | 20 | -0.00399 | 0.000724 | -0.00399 | 0.000724 | -0.00399 | 0.000724 | -0.0019 | 0.00023 |  |  |
| rs5760495 | 22 | 0.00156 | 0.000239 | 0.00156 | 0.000239 | 0.00156 | 0.000239 | 0.002584 | 0.000457 | rs5751901 | |
| rs36086195 | 1 | 0.001839 | 0.000229 | 0.001839 | 0.000229 | 0.001839 | 0.000229 | 0.001341 | 0.000242 |  |  |
| rs841572 | 1 | 0.002805 | 0.000231 | 0.002805 | 0.000231 | 0.002805 | 0.000231 | -0.00208 | 0.000268 |  |  |
| rs1848837 | 1 | -0.00148 | 0.00025 | -0.00148 | 0.00025 | -0.00148 | 0.00025 | 0.003306 | 0.000512 |  |  |
| rs629301 | 1 | 0.00158 | 0.000272 | 0.00158 | 0.000272 | 0.00158 | 0.000272 | 0.005811 | 0.000396 |  |  |
| rs10737767 | 1 | 0.001402 | 0.000231 | 0.001402 | 0.000231 | 0.001402 | 0.000231 | -0.00193 | 0.000259 |  |  |
| rs1434282 | 1 | 0.002059 | 0.000254 | 0.002059 | 0.000254 | 0.002059 | 0.000254 | 0.002472 | 0.000348 |  |  |
| rs4506486 | 1 | 0.001539 | 0.000231 | 0.001539 | 0.000231 | 0.001539 | 0.000231 | 0.001851 | 0.000263 |  |  |
| rs1497826 | 1 | 0.001792 | 0.000235 | 0.001792 | 0.000235 | 0.001792 | 0.000235 | 0.003259 | 0.000526 |  |  |
| rs6667260 | 1 | -0.0016 | 0.000227 | -0.0016 | 0.000227 | -0.0016 | 0.000227 | -0.00132 | 0.000229 |  |  |
| rs12467820 | 2 | 0.001244 | 0.000227 | 0.001244 | 0.000227 | 0.001244 | 0.000227 | 0.001792 | 0.000334 |  |  |
| rs10193807 | 2 | 0.001816 | 0.00031 | 0.001816 | 0.00031 | 0.001816 | 0.00031 | -0.00188 | 0.00025 |  |  |
| rs6545412 | 2 | 0.002016 | 0.000316 | 0.002016 | 0.000316 | 0.002016 | 0.000316 | -0.00131 | 0.000232 |  |  |
| rs12468920 | 2 | 0.001388 | 0.000246 | 0.001388 | 0.000246 | 0.001388 | 0.000246 | 0.001577 | 0.000232 |  |  |
| rs6576983 | 2 | -0.0035 | 0.000232 | -0.0035 | 0.000232 | -0.0035 | 0.000232 | -0.00233 | 0.000273 |  |  |
| rs895418 | 2 | 0.002143 | 0.000228 | 0.002143 | 0.000228 | 0.002143 | 0.000228 | 0.00211 | 0.000357 |  |  |
| rs13389219 | 2 | -0.00139 | 0.000231 | -0.00139 | 0.000231 | -0.00139 | 0.000231 | -0.00216 | 0.000228 |  |  |
| rs12613807 | 2 | 0.001483 | 0.000228 | 0.001483 | 0.000228 | 0.001483 | 0.000228 | -0.00338 | 0.00042 |  |  |
| rs7559013 | 2 | 0.002221 | 0.000337 | 0.002221 | 0.000337 | 0.002221 | 0.000337 | -0.00273 | 0.000235 |  |  |
| rs838717 | 2 | -0.00375 | 0.000229 | -0.00375 | 0.000229 | -0.00375 | 0.000229 | -0.0051 | 0.000474 |  |  |
| rs10197122 | 2 | 0.002422 | 0.000439 | 0.002422 | 0.000439 | 0.002422 | 0.000439 | 0.003035 | 0.000238 |  |  |
| rs60624480 | 2 | -0.00218 | 0.000236 | -0.00218 | 0.000236 | -0.00218 | 0.000236 | -0.00199 | 0.000321 |  |  |
| rs35154162 | 3 | -0.00191 | 0.000268 | -0.00191 | 0.000268 | -0.00191 | 0.000268 | -0.00162 | 0.000284 |  |  |
| rs1801282 | 3 | -0.00332 | 0.000347 | -0.00332 | 0.000347 | -0.00332 | 0.000347 | -0.00264 | 0.000305 |  |  |
| rs11709284 | 3 | 0.001386 | 0.000227 | 0.001386 | 0.000227 | 0.001386 | 0.000227 | 0.001886 | 0.000265 |  |  |
| rs12485738 | 3 | -0.0017 | 0.000234 | -0.0017 | 0.000234 | -0.0017 | 0.000234 | -0.00187 | 0.000227 |  |  |
| rs16853573 | 3 | 0.004122 | 0.00043 | 0.004122 | 0.00043 | 0.004122 | 0.00043 | 0.001964 | 0.000298 |  |  |
| rs2271494 | 3 | 0.001836 | 0.000229 | 0.001836 | 0.000229 | 0.001836 | 0.000229 | 0.001641 | 0.000229 |  |  |
| rs73186030 | 3 | -0.01767 | 0.000335 | -0.01767 | 0.000335 | -0.01767 | 0.000335 | -0.0017 | 0.000229 |  |  |
| rs28519617 | 3 | 0.002542 | 0.000256 | 0.002542 | 0.000256 | 0.002542 | 0.000256 | -0.00276 | 0.000407 |  |  |
| rs3773912 | 3 | -0.00128 | 0.000227 | -0.00128 | 0.000227 | -0.00128 | 0.000227 | -0.0016 | 0.000261 |  |  |
| rs7688574 | 4 | 0.001386 | 0.000235 | 0.001386 | 0.000235 | 0.001386 | 0.000235 | 0.001311 | 0.000232 |  |  |
| rs2713844 | 4 | -0.00165 | 0.000267 | -0.00165 | 0.000267 | -0.00165 | 0.000267 | 0.002855 | 0.000234 |  |  |
| rs2388993 | 4 | -0.00131 | 0.00023 | -0.00131 | 0.00023 | -0.00131 | 0.00023 | -0.00199 | 0.000238 |  |  |
| rs4320103 | 4 | 0.00452 | 0.000591 | 0.00452 | 0.000591 | 0.00452 | 0.000591 | -0.00178 | 0.000269 |  |  |
| rs10942734 | 5 | 0.001531 | 0.000229 | 0.001531 | 0.000229 | 0.001531 | 0.000229 | 0.005728 | 0.000722 |  |  |
| rs6897362 | 5 | -0.00133 | 0.000227 | -0.00133 | 0.000227 | -0.00133 | 0.000227 | -0.00146 | 0.000229 |  |  |
| rs251391 | 5 | 0.001483 | 0.000261 | 0.001483 | 0.000261 | 0.001483 | 0.000261 | -0.00185 | 0.000229 |  |  |
| rs12519940 | 5 | -0.00231 | 0.000255 | -0.00231 | 0.000255 | -0.00231 | 0.000255 | -0.00216 | 0.000262 |  |  |
| rs7742369 | 6 | 0.003858 | 0.000298 | 0.003858 | 0.000298 | 0.003858 | 0.000298 | 0.001554 | 0.000234 | rs10807138 | |
| rs1214761 | 6 | -0.00133 | 0.000243 | -0.00133 | 0.000243 | -0.00133 | 0.000243 | -0.00549 | 0.000433 |  |  |
| rs4263551 | 6 | -0.00346 | 0.000227 | -0.00346 | 0.000227 | -0.00346 | 0.000227 | -0.00164 | 0.000291 |  |  |
| rs4946137 | 6 | 0.001687 | 0.000232 | 0.001687 | 0.000232 | 0.001687 | 0.000232 | 0.00825 | 0.000941 |  |  |
| rs9388399 | 6 | -0.00209 | 0.000245 | -0.00209 | 0.000245 | -0.00209 | 0.000245 | 0.001603 | 0.00023 |  |  |
| rs7756870 | 6 | -0.00205 | 0.000245 | -0.00205 | 0.000245 | -0.00205 | 0.000245 | -0.00164 | 0.000286 |  |  |
| rs1763519 | 6 | -0.00253 | 0.000232 | -0.00253 | 0.000232 | -0.00253 | 0.000232 | -0.00265 | 0.000408 |  |  |
| rs3857708 | 7 | 0.001881 | 0.000249 | 0.001881 | 0.000249 | 0.001881 | 0.000249 | 0.002446 | 0.000451 |  |  |
| rs4718271 | 7 | 0.002856 | 0.000228 | 0.002856 | 0.000228 | 0.002856 | 0.000228 | -0.00176 | 0.000229 |  |  |
| rs7786368 | 7 | -0.0019 | 0.00023 | -0.0019 | 0.00023 | -0.0019 | 0.00023 | -0.00192 | 0.00026 |  |  |
| rs2283038 | 7 | -0.00208 | 0.000268 | -0.00208 | 0.000268 | -0.00208 | 0.000268 | -0.00958 | 0.00073 |  |  |
| rs4240624 | 8 | 0.005811 | 0.000396 | 0.005811 | 0.000396 | 0.005811 | 0.000396 | -0.00328 | 0.000316 |  |  |
| rs36104352 | 8 | 0.002472 | 0.000348 | 0.002472 | 0.000348 | 0.002472 | 0.000348 | -0.00432 | 0.000771 |  |  |
| rs28601761 | 8 | -0.00131 | 0.000232 | -0.00131 | 0.000232 | -0.00131 | 0.000232 | -0.00202 | 0.000262 |  |  |
| rs4740965 | 9 | -0.00233 | 0.000273 | -0.00233 | 0.000273 | -0.00233 | 0.000273 | 0.002638 | 0.000396 |  |  |
| rs11144001 | 9 | -0.00216 | 0.000228 | -0.00216 | 0.000228 | -0.00216 | 0.000228 | 0.001541 | 0.000244 |  |  |
| rs12337706 | 9 | -0.0051 | 0.000474 | -0.0051 | 0.000474 | -0.0051 | 0.000474 | 0.001733 | 0.000323 |  |  |
| rs10819178 | 9 | 0.003035 | 0.000238 | 0.003035 | 0.000238 | 0.003035 | 0.000238 | 0.002627 | 0.000488 |  |  |
| rs498490 | 10 | -0.00264 | 0.000305 | -0.00264 | 0.000305 | -0.00264 | 0.000305 | -0.00276 | 0.000367 |  |  |
| rs3011642 | 10 | 0.001886 | 0.000265 | 0.001886 | 0.000265 | 0.001886 | 0.000265 | -0.00233 | 0.000303 |  |  |
| rs2377965 | 10 | -0.00187 | 0.000227 | -0.00187 | 0.000227 | -0.00187 | 0.000227 | 0.01164 | 0.000817 |  |  |
| rs1262217 | 10 | 0.001964 | 0.000298 | 0.001964 | 0.000298 | 0.001964 | 0.000298 | -0.00183 | 0.000271 |  |  |
| rs9420589 | 10 | 0.001641 | 0.000229 | 0.001641 | 0.000229 | 0.001641 | 0.000229 | 0.008869 | 0.000734 |  |  |
| rs2274224 | 10 | -0.0017 | 0.000229 | -0.0017 | 0.000229 | -0.0017 | 0.000229 | -0.00225 | 0.000303 |  |  |
| rs2296436 | 10 | -0.00276 | 0.000407 | -0.00276 | 0.000407 | -0.00276 | 0.000407 | -0.00154 | 0.000229 |  |  |
| rs2419886 | 10 | -0.0016 | 0.000261 | -0.0016 | 0.000261 | -0.0016 | 0.000261 | -0.00301 | 0.000507 |  |  |
| rs7947953 | 11 | 0.001311 | 0.000232 | 0.001311 | 0.000232 | 0.001311 | 0.000232 | 0.001657 | 0.000262 |  |  |
| rs11511848 | 11 | 0.002855 | 0.000234 | 0.002855 | 0.000234 | 0.002855 | 0.000234 | -0.0021 | 0.000237 |  |  |
| rs12363232 | 11 | -0.00199 | 0.000238 | -0.00199 | 0.000238 | -0.00199 | 0.000238 | 0.001276 | 0.000236 |  | rs35624992 |
| rs2298615 | 11 | -0.00178 | 0.000269 | -0.00178 | 0.000269 | -0.00178 | 0.000269 | -0.00141 | 0.000241 |  |  |
| rs302650 | 11 | -0.00185 | 0.000229 | -0.00185 | 0.000229 | -0.00185 | 0.000229 | -0.00131 | 0.000248 |  |  |
| rs11218721 | 11 | 0.001554 | 0.000234 | 0.001554 | 0.000234 | 0.001554 | 0.000234 | 0.004561 | 0.000695 |  |  |
| rs2855799 | 11 | -0.00164 | 0.000291 | -0.00164 | 0.000291 | -0.00164 | 0.000291 | -0.00359 | 0.000527 |  |  |
| rs6580981 | 12 | -0.00176 | 0.000229 | -0.00176 | 0.000229 | -0.00176 | 0.000229 | -0.00225 | 0.000272 |  |  |
| rs11172256 | 12 | -0.00192 | 0.00026 | -0.00192 | 0.00026 | -0.00192 | 0.00026 | 0.002785 | 0.00024 |  |  |
| rs7319447 | 13 | -0.00328 | 0.000316 | -0.00328 | 0.000316 | -0.00328 | 0.000316 | 0.001374 | 0.000228 |  |  |
| rs12583851 | 13 | -0.00202 | 0.000262 | -0.00202 | 0.000262 | -0.00202 | 0.000262 | -0.00264 | 0.000229 |  |  |
| rs8011945 | 14 | 0.002638 | 0.000396 | 0.002638 | 0.000396 | 0.002638 | 0.000396 | 0.002444 | 0.000347 |  |  |
| rs17718872 | 14 | 0.001733 | 0.000323 | 0.001733 | 0.000323 | 0.001733 | 0.000323 | 0.004854 | 0.000291 |  |  |
| rs35852840 | 14 | 0.002627 | 0.000488 | 0.002627 | 0.000488 | 0.002627 | 0.000488 | 0.001871 | 0.000283 |  |  |
| rs7144433 | 14 | -0.00276 | 0.000367 | -0.00276 | 0.000367 | -0.00276 | 0.000367 | 0.001721 | 0.000294 |  |  |
| rs17127600 | 14 | -0.00233 | 0.000303 | -0.00233 | 0.000303 | -0.00233 | 0.000303 | -0.00161 | 0.00025 |  |  |
| rs147233090 | 15 | 0.008869 | 0.000734 | 0.008869 | 0.000734 | 0.008869 | 0.000734 | -0.00185 | 0.000277 |  |  |
| rs11632520 | 15 | -0.00225 | 0.000303 | -0.00225 | 0.000303 | -0.00225 | 0.000303 | 0.003908 | 0.00044 |  |  |
| rs12911748 | 15 | -0.00154 | 0.000229 | -0.00154 | 0.000229 | -0.00154 | 0.000229 | -0.0023 | 0.000257 |  |  |
| rs11071896 | 15 | 0.001657 | 0.000262 | 0.001657 | 0.000262 | 0.001657 | 0.000262 | -0.0086 | 0.000762 |  |  |
| rs34066945 | 15 | -0.0021 | 0.000237 | -0.0021 | 0.000237 | -0.0021 | 0.000237 | 0.002144 | 0.000384 |  |  |
| rs11629876 | 15 | -0.00141 | 0.000241 | -0.00141 | 0.000241 | -0.00141 | 0.000241 | 0.001359 | 0.000232 |  |  |
| rs2745205 | 16 | -0.00131 | 0.000248 | -0.00131 | 0.000248 | -0.00131 | 0.000248 | 0.001411 | 0.000253 |  |  |
| rs16945716 | 16 | -0.00359 | 0.000527 | -0.00359 | 0.000527 | -0.00359 | 0.000527 | -0.00569 | 0.000369 |  |  |
| rs1858800 | 16 | 0.002785 | 0.00024 | 0.002785 | 0.00024 | 0.002785 | 0.00024 | 0.001276 | 0.000232 |  |  |
| rs35610022 | 16 | 0.001374 | 0.000228 | 0.001374 | 0.000228 | 0.001374 | 0.000228 | 0.004958 | 0.000595 |  |  |
| rs12918968 | 16 | -0.00264 | 0.000229 | -0.00264 | 0.000229 | -0.00264 | 0.000229 | 0.006755 | 0.000455 |  |  |
| rs12600694 | 17 | 0.001871 | 0.000283 | 0.001871 | 0.000283 | 0.001871 | 0.000283 | -0.00876 | 0.000841 |  |  |
| rs66634575 | 17 | 0.001721 | 0.000294 | 0.001721 | 0.000294 | 0.001721 | 0.000294 | -0.00157 | 0.000277 |  |  |
| rs7221118 | 17 | -0.00185 | 0.000277 | -0.00185 | 0.000277 | -0.00185 | 0.000277 | -0.00146 | 0.00026 |  |  |
| rs9910998 | 17 | 0.003908 | 0.00044 | 0.003908 | 0.00044 | 0.003908 | 0.00044 | 0.002122 | 0.000263 |  |  |
| rs2270114 | 17 | -0.0023 | 0.000257 | -0.0023 | 0.000257 | -0.0023 | 0.000257 | 0.004687 | 0.0007 |  |  |
| rs10852764 | 17 | 0.002144 | 0.000384 | 0.002144 | 0.000384 | 0.002144 | 0.000384 | 0.008099 | 0.00057 |  |  |
| rs55722786 | 18 | 0.001411 | 0.000253 | 0.001411 | 0.000253 | 0.001411 | 0.000253 | -0.00136 | 0.000241 |  |  |
| rs1672991 | 19 | 0.006755 | 0.000455 | 0.006755 | 0.000455 | 0.006755 | 0.000455 | -0.00399 | 0.000724 |  |  |
| rs7248167 | 19 | -0.00157 | 0.000277 | -0.00157 | 0.000277 | -0.00157 | 0.000277 | -0.00171 | 0.000248 |  |  |
| rs928760 | 21 | -0.00171 | 0.000248 | -0.00171 | 0.000248 | -0.00171 | 0.000248 | 0.001459 | 0.000258 |  |  |
| rs219781 | 21 | 0.001459 | 0.000258 | 0.001459 | 0.000258 | 0.001459 | 0.000258 | 0.00156 | 0.000239 |  |  |

eFigure 1. Principles of mendelian randomization study for serum magnesium and calcium concentrations, and epilepsy risk.


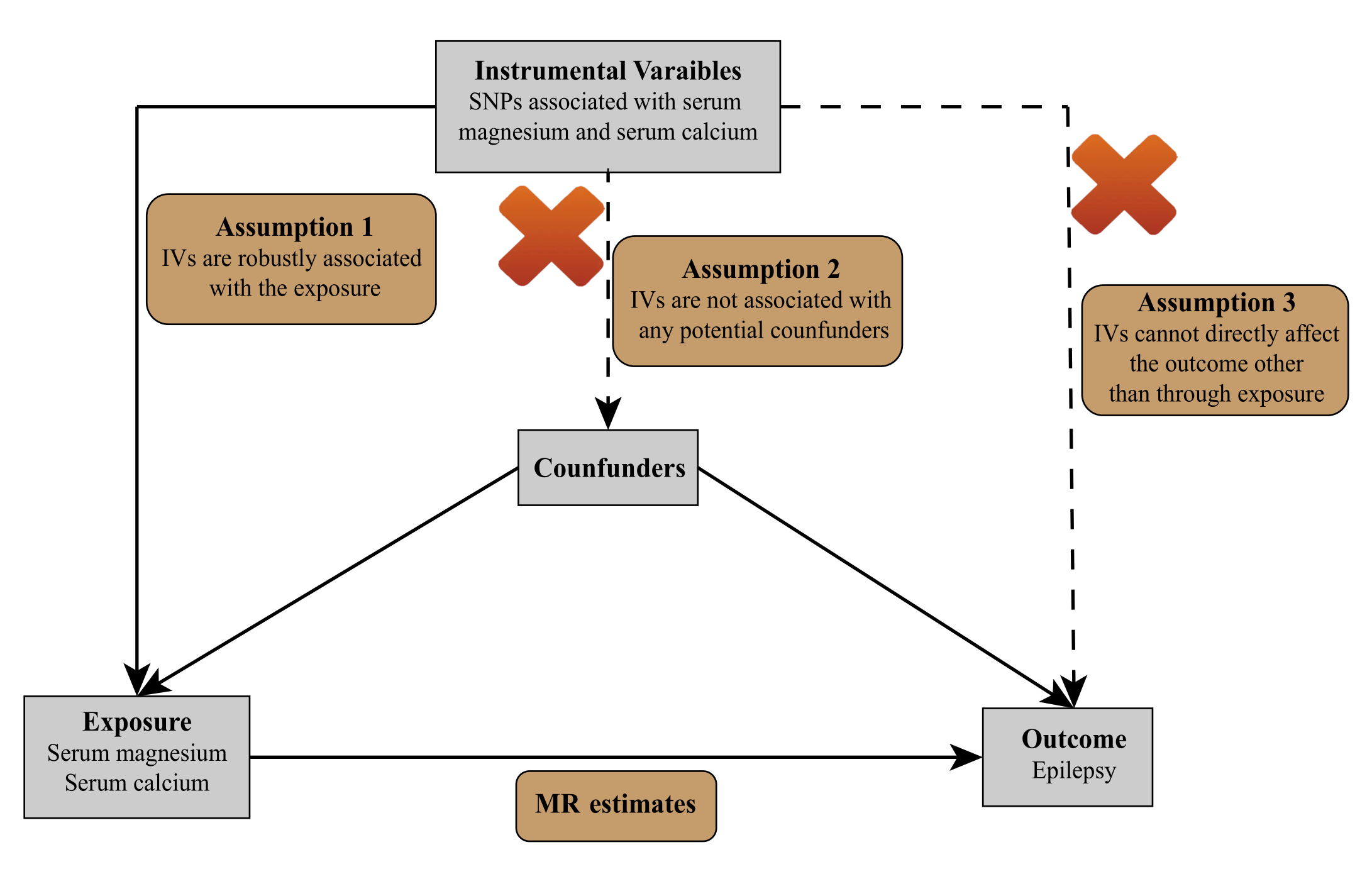


eFigure 2. Association between genetically predicted serum magnesium and calcium concentrations and epilepsy in FinnGen,


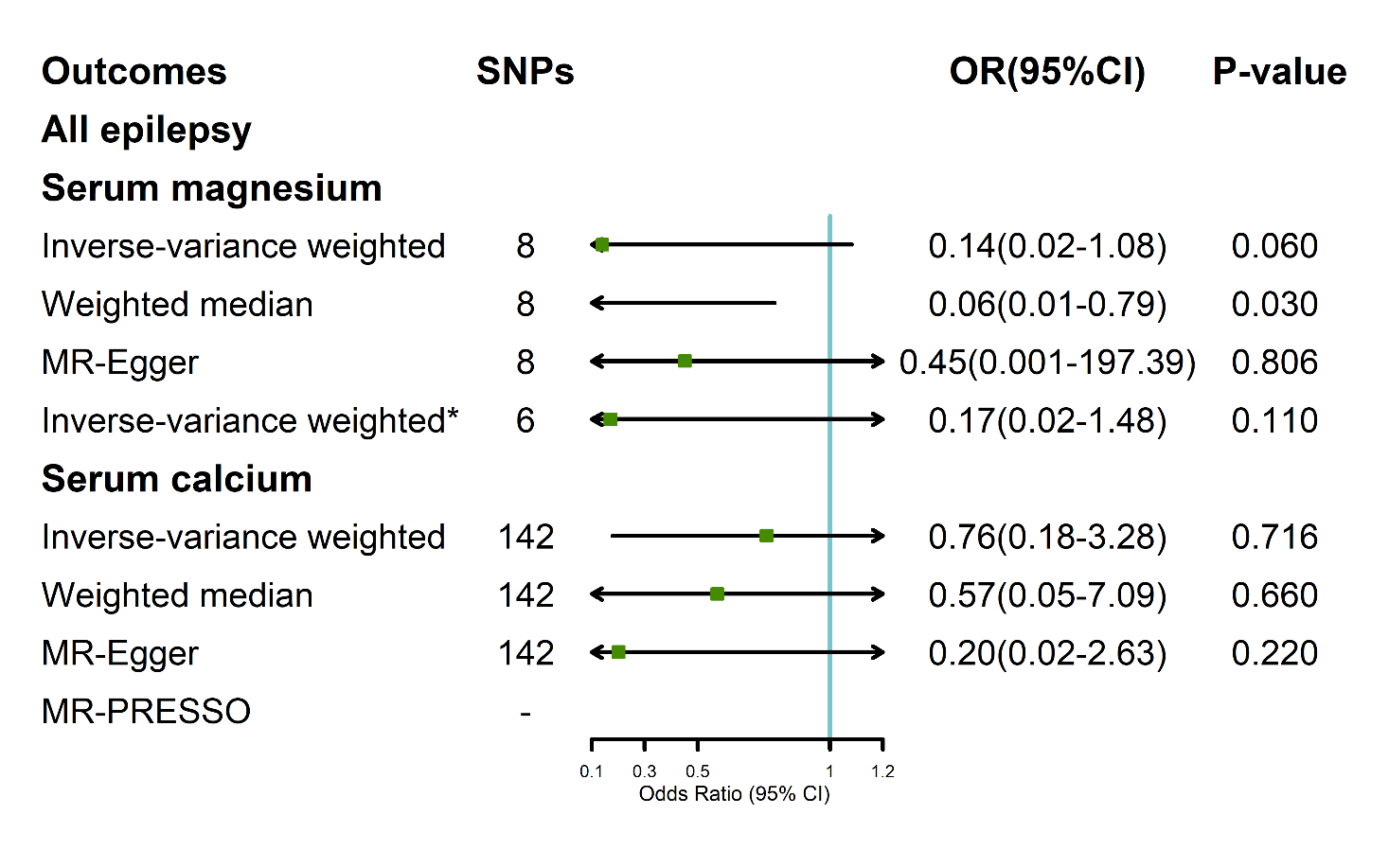


^*^Inverse-variance weighted excluding two genetic variants (rs3740393 and rs6746896) in or near known magnesium transport genes and associated with serum magnesium after applying a Bonferroni correction for the number of genetic variants assessed in each region.

**International League Against Epilepsy Consortium on Complex Epilepsies members Members listed in alphabetical order:**

Bassel Abou-Khalil1, Pauls Auce2, 3, Andreja Avbersek4, Melanie Bahlo5-7, David J Balding8, 9, Thomas Bast10, 11, Larry Baum12-14, Albert J Becker15, Felicitas Becker16, 17 Bianca Berghuis18, Samuel F Berkovic19, Katja E Boysen19, Jonathan P Bradfield20, 21, Lawrence C Brody22, Russell J Buono20, 23, 24, Ellen Campbell25, Gregory D Cascino26, Claudia B Catarino4, Gianpiero L Cavalleri27, 28, Stacey S Cherny13, 29, Krishna Chinthapalli4, Alison J Coffey30, Alastair Compston31, Antonietta Coppola32, 33, Patrick Cossette34, John J Craig35, Gerrit-Jan de Haan36, Peter De Jonghe37, 38, Carolien G F de Kovel39, Norman Delanty27, 28, 40, Chantal Depondt41, Orrin Devinsky42, Dennis J Dlugos43, Colin P Doherty28, 44, Christian E Elger45, Johan G Eriksson46, Thomas N Ferraro23, 47, Martha Feucht48, Ben Francis49, Andre Franke50, Jacqueline A French51, Saskia Freytag5, Verena Gaus52, Eric B Geller53, Christian Gieger54, 55, Tracy Glauser56, Simon Glynn57, David B Goldstein58, 59, Hongsheng Gui13, Youling Guo13, Kevin F Haas1, Hakon Hakonarson20, 60, Kerstin Hallmann45, 61, Sheryl Haut62, Erin L Heinzen58, 59, Ingo Helbig43, 63, Christian Hengsbach16, Helle Hjalgrim64, 65, Michele Iacomino33, Andrés Ingason66, Jennifer Jamnadas-Khoda4, 67, Michael R Johnson68, Reetta Kälviäinen69, 70, Anne-Mari Kantanen69, Dalia Kasperavičiūte4, Dorothee Kasteleijn-Nolst Trenite39, Heidi E Kirsch71, Robert C Knowlton72, Bobby P C Koeleman39, Roland Krause73, Martin Krenn74, Wolfram S Kunz45, Ruben Kuzniecky75, Patrick Kwan12, 76, 77, Dennis Lal78, Yu-Lung Lau79, Anna-Elina Lehesjoki80, Holger Lerche16, Costin Leu4, 78, 81, Wolfgang Lieb82, Dick Lindhout36, 39, Warren D Lo83, Iscia Lopes-Cendes84, 85, Daniel H Lowenstein71, Alberto Malovini86, Anthony G Marson2, Thomas Mayer87, Mark McCormack27, James L Mills88, Nasir Mirza2, Martina Moerzinger48, Rikke S Møller64, 65, 89, Anne M Molloy90, Hiltrud Muhle63, Mark Newton91, Ping-Wing Ng92, Markus M Nöthen93, Peter Nürnberg94, Terence J O’Brien76, 77, Karen L Oliver19, Aarno Palotie95, 96, Faith Pangilinan22, Sarah Peter73, Slavé Petrovski76, 97, Annapurna Poduri98, Michael Privitera99, Rodney Radtke100, Sarah Rau16, Philipp S Reif101, 102, Eva M Reinthaler74, Felix Rosenow101, 102, Josemir W Sander4, 36, 103, Thomas Sander52, 94, Theresa Scattergood104, Steven C Schachter105, Christoph J Schankin106, Ingrid E Scheffer19, 107, Bettina Schmitz52, Susanne Schoch15, Pak C Sham13, Jerry J Shih108, Graeme J Sills2, Sanjay M Sisodiya4, 103, Lisa Slattery109, Alexander Smith78, David F Smith3, Michael C Smith110, Philip E Smith111, Anja C M Sonsma39, Doug Speed8, 112, Michael R Sperling113, Bernhard J Steinhoff10, Ulrich Stephani63, Remi Stevelink39, Konstantin Strauch114, 115, Pasquale Striano116, Hans Stroink117, Rainer Surges45, K Meng Tan76, Liu Lin Thio118, G Neil Thomas119, Marian Todaro76, Rossana Tozzi120, Maria S Vari116, Eileen P G Vining121, Frank Visscher122, Sarah von Spiczak63, Nicole M Walley58, 123, Yvonne G Weber16,126, Zhi Wei124, Judith Weisenberg118, Christopher D Whelan27, Peter Widdess-Walsh53, Markus Wolff125, Stefan Wolking16, Wanling Yang79, Federico Zara33, Fritz Zimprich74

1. Vanderbilt University Medical Center, Nashville, TN 37232, USA.

2. Department of Molecular and Clinical Pharmacology, University of Liverpool, Liverpool L69 3GL, UK.

3. The Walton Centre NHS Foundation Trust, Liverpool L9 7LJ, UK.

4. Department of Clinical and Experimental Epilepsy, UCL Institute of Neurology, Queen Square, London WC1N 3BG, UK.

5. Population Health and Immunity Divison, The Walter and Eliza Hall Institute of Medical Research, Parkville 3052, Australia.

6. Department of Biology, University of Melbourne, Parkville 3010, Australia.

7. School of Mathematics and Statistics, University of Melbourne, Parkville 3010, Australia.

8. UCL Genetics Institute, University College London, London WC1E 6BT, UK.

9. Melbourne Integrative Genomics, University of Melbourne, Parkville 3052, Australia.

10. Epilepsy Center Kork, Kehl-Kork 77694, Germany.

11. Medical Faculty of the University of Freiburg, Freiburg 79085, Germany.

12. Centre for Genomic Sciences, The University of Hong Kong, Hong Kong.

13. Department of Psychiatry, The University of Hong Kong, Hong Kong.

14. The State Key Laboratory of Brain and Cognitive Sciences, University of Hong Kong, Hong Kong, China.

15. Section for Translational Epilepsy Research, Department of Neuropathology, University of Bonn Medical Center, Bonn 53105, Germany.

16. Department of Neurology and Epileptology, Hertie Institute for Clinical Brain Research, University of Tübingen, Tübingen 72076, Germany.

17. Department of Neurology, University of Ulm, Ulm 89081, Germany.

18. Stichting Epilepsie Instellingen Nederland (SEIN), Zwolle 8025 BV, The Netherlands.

19. Epilepsy Research Centre, University of Melbourne, Austin Health, Heidelberg 3084, Australia.

20. Center for Applied Genomics, The Children's Hospital of Philadelphia, Philadelphia, PA 19104, USA.

21. Quantinuum Research LLC, San Diego, CA 92101, USA.

22. National Human Genome Research Institute, National Institutes of Health, Bethesda, MD 20892, USA.

23. Department of Biomedical Sciences, Cooper Medical School of Rowan University Camden, NJ 08103, USA.

24. Department of Neurology, Thomas Jefferson University Hospital, Philadelphia, PA 19107, USA.

25. Belfast Health and Social Care Trust, Belfast BT9 7AB, UK.

26. Division of Epilepsy, Department of Neurology, Mayo Clinic, Rochester, MN 55902, USA.

27. Department of Molecular and Cellular Therapeutics, The Royal College of Surgeons in Ireland, Dublin 2, Ireland.

28. The FutureNeuro Research Centre, Dublin 2, Ireland.

29. Department of Epidemiology and Preventive Medicine, School of Public Health, Sackler Faculty of Medicine, Tel Aviv University, Tel Aviv 6997801, Israel.

30. The Wellcome Trust Sanger Institute, Hinxton, Cambridge CB10 1SA, UK.

31. Department of Clinical Neurosciences, Cambridge Biomedical Campus, Cambridge CB2 0SL, UK.

32. Department of Neuroscience, Reproductive and Odontostomatological Sciences, University Federico II, Naples 80138, Italy.

33. Laboratory of Neurogenetics and Neurosciences, Institute G. Gaslini, Genova 16148, Italy.

34. Department of Neurosciences, Université de Montréal, Montréal, CA 26758, Canada.

35. Department of Neurology, Royal Victoria Hospital, Belfast Health and Social Care Trust, Grosvenor Road, Belfast BT12 6BA, UK.

36. Stichting Epilepsie Instellingen Nederland (SEIN), Heemstede 2103 SW, The Netherlands.

37. Neurogenetics Group, Center for Molecular Neurology, VIB and Laboratory of Neurogenetics, Institute Born-Bunge, University of Antwerp, Antwerp 2610, Belgium.

38. Department of Neurology, Antwerp University Hospital, Edegem 2650, Belgium.

39. Department of Genetics, University Medical Center Utrecht, Utrecht 3584 CX, The Netherlands.

40. Division of Neurology, Beaumont Hospital, Dublin D09 FT51, Ireland.

41. Department of Neurology, Hôpital Erasme, Université Libre de Bruxelles, Bruxelles 1070, Belgium.

42. Comprehensive Epilepsy Center, New York University School of Medicine, New York, NY 10016, USA.

43. Department of Neurology, The Children's Hospital of Philadelphia, Philadelphia, PA 19104, USA.

44. Neurology Department, St. James’s Hospital, Dublin D03 VX82, Ireland.

45. Department of Epileptology, University of Bonn Medical Centre, Bonn 53127, Germany.

46. Department of General Practice and Primary Health Care, University of Helsinki and Helsinki University Hospital, Helsinki 0014, Finland.

47. Department of Pharmacology and Psychiatry, University of Pennsylvania Perlman School of Medicine, Philadelphia, PA 19104, USA.

48. Department of Pediatrics and Neonatology, Medical University of Vienna, Vienna 1090, Austria.

49. Department of Biostatistics, University of Liverpool, Liverpool L69 3GL, UK.

50. Institute of Clinical Molecular Biology, Christian-Albrechts-University of Kiel, University Hospital Schleswig Holstein, Kiel 24105, Germany.

51. Department of Neurology, NYU School of Medicine, New York City, NY 10003, USA.

52. Department of Neurology, Charité Universitaetsmedizin Berlin, Campus Virchow-Clinic, Berlin 13353, Germany.

53. Institute of Neurology and Neurosurgery at St. Barnabas, Livingston, NJ 07039, USA.

54. Research Unit of Molecular Epidemiology, Helmholtz Zentrum München - German Research Center for Environmental Health, Neuherberg D-85764, Germany.

55. Institute of Epidemiology, Helmholtz Zentrum München - German Research Center for Environmental Health, Neuherberg D-85764, Germany.

56. Comprehensive Epilepsy Center, Division of Neurology, Cincinnati Children's Hospital Medical Center, Cincinnati, OH 45229, USA.

57. Department of Neurology, University of Michigan, Ann Arbor, MI 48109, USA.

58. Center for Human Genome Variation, Duke University School of Medicine, Durham, NC 27710, USA.

59. Institute for Genomic Medicine, Columbia University Medical Center, New York, NY 10032, USA.

60. Division of Human Genetics, Department of Pediatrics, The Perelman School of Medicine, University of Pennsylvania, Philadelphia, PA 19104, USA.

61. Life and Brain Center, University of Bonn Medical Center, Bonn 53127, Germany.

62. Montefiore Medical Center, Bronx, NY 10467, USA.

63. Department of Neuropediatrics, University Medical Center Schleswig-Holstein (UKSH), Kiel 24105, Germany.

64. Danish Epilepsy Centre, Dianalund 4293, Denmark.

65. Institute of Regional Health Services Research, University of Southern Denmark, Odense 5000, Denmark.

66. deCODE genetics, Reykjavik IS-101, Iceland.

67. Department of Psychiatry and Applied Psychology, Institute of Mental Health University of Nottingham, Nottingham NG7 2TU, UK.

68. Faculty of Medicine, Imperial College London, London SW7 2AZ, UK.

69. Kuopio Epilepsy Center, Neurocenter, Kuopio University Hospital, Kuopio 70029, Finland.

70. Institute of Clinical Medicine, University of Eastern Finland, Kuopio 70029, Finland.

71. Department of Neurology, University of California, San Francisco, CA 94143, USA.

72. University of Alabama Birmingham, Department of Neurology, Birmingham, AL 35233, USA.

73. Luxembourg Centre for Systems Biomedicine, University of Luxembourg, Esch-sur-Alzette L-4362, Luxembourg.

74. Department of Neurology, Medical University of Vienna, Vienna 1090, Austria.

75. Department of Neurology, Zucker-Hofstra Northwell School of Medicine, NY 10075, USA.

76. Department of Medicine, University of Melbourne, Royal Melbourne Hospital, Parkville 3050, Australia.

77. Department of Neuroscience, Central Clinical School, Monash University, Melbourne 3004, Australia.

78. Stanley Center for Psychiatric Research, Broad Institute of Harvard and M.I.T., Cambridge, MA 02142, USA.

79. Department of Paediatrics and Adolescent Medicine, The University of Hong Kong, Hong Kong.

80. Folkhälsan Research Center and Medical Faculty, University of Helsinki, Helsinki 00290, Finland.

81. Genomic Medicine Institute, Lerner Research Institute, Cleveland Clinic, Cleveland, OH 44195, USA.

82. Institut für Epidemiologie Christian-Albrechts-Universität zu Kiel, Kiel 24105, Germany.

83. Department of Pediatrics and Neurology, Ohio State University and Nationwide Children's Hospital, Columbus, OH 43205, USA.

84. Department of Medical Genetics, School of Medical Sciences, University of Campinas

(UNICAMP), Campinas, SP 13083-887, Brazil.

85. Brazilian Institute of Neuroscience and Neurotechnology (BRAINN), Campinas, SP 13083-970, Brazil.

86. Istituti Clinici Scientifici Maugeri, Pavia 27100, Italy.

87. Epilepsy Center Kleinwachau, Radeberg 01454, Germany.

88. Division of Intramural Population Health Research, Eunice Kennedy Shriver National Institute of Child Health and Human Development, National Institutes of Health, Bethesda, MD 20892, USA.

89. Wilhelm Johannsen Centre for Functional Genome Research, Copenhagen DK-2200, Denmark.

90. School of Medicine, Trinity College Dublin, Dublin 2, Ireland.

91. Department of Neurology, Austin Health, Heidelberg 3084, Australia.

92. United Christian Hospital, Hong Kong.

93. Institute of Human Genetics, University of Bonn Medical Center, Bonn 53127, Germany.

94. Cologne Center for Genomics, University of Cologne, Cologne 50931, Germany.

95. Institute for Molecular Medicine Finland (FIMM), University of Helsinki, Helsinki 0014, Finland.

96. The Broad Institute of M.I.T. and Harvard, Cambridge, MA 02142, USA.

97. AstraZeneca Centre for Genomics Research, Precision Medicine and Genomics, IMED Biotech Unit, AstraZeneca, Cambridge CB2 0AA, UK.

98. Department of Neurology, Boston Children's Hospital, Harvard Medical School, Boston, MA 02115, USA.

99. Department of Neurology, Neuroscience Institute, University of Cincinnati Medical Center, Cincinnati, OH 45220, USA.

100. Department of Neurology, Duke University School of Medicine, Durham, NC 27710, USA.

101. Epilepsy-Center Hessen, Department of Neurology, University Medical Center Giessen and Marburg, Marburg, Germany and Philipps-University Marburg, Marburg 35043, Germany.

102. Epilepsy Center Frankfurt Rhine-Main, Center of Neurology and Neurosurgery, Goethe University Frankfurt, Frankfurt 60528, Germany.

103. Chalfont Centre for Epilepsy, Chalfont-St-Peter, Buckinghamshire SL9 0RJ, UK.

104. Department of Endocrinology, Hospital of The University of Pennsylvania, Philadelphia, PA 19104, USA.

105. Departments of Neurology, Beth Israel Deaconess Medical Center, Massachusetts General Hospital, and Harvard Medical School, Boston, MA 02215, USA.

106. Department of Neurology, Inselspital, Bern University Hospital, University of Bern, Bern 3010, Switzerland.

107. Department of Neurology, Royal Children's Hospital, Parkville 3052, Australia.

108. Department of Neurosciences, University of California, San Diego, La Jolla, CA 92037, USA.

109. The Royal College of Surgeons in Ireland, Dublin D02 YN77, Ireland. .

110. Rush University Medical Center, Chicago, IL 60612, USA.

111. Department of Neurology, Alan Richens Epilepsy Unit, University Hospital of Wales, Cardiff CF14 4XW, UK.

112. Aarhus Institute of Advanced Studies (AIAS), Aarhus University, 8000 Aarhus, Denmark.

113. Department of Neurology and Comprehensive Epilepsy Center, Thomas Jefferson University, Philadelphia, PA 19107, USA.

114. Institute of Genetic Epidemiology, Helmholtz Zentrum München - German Research Center for Environmental Health, Neuherberg D-85764, Germany.

115. Chair of Genetic Epidemiology, IBE, Faculty of Medicine, LMU Munich 80539, Germany.

116. Pediatric Neurology and Muscular Diseases Unit, Department of Neurosciences, Rehabilitation, Ophthalmology, Genetics, Maternal and Child Health, G. Gaslini Institute, University of Genoa, Genova 16148, Italy.

117. CWZ Hospital, 6532 SZ Nijmegen, The Netherlands.

118. Department of Neurology, Washington University School of Medicine, St. Louis, MO 63110, USA.

119.Institute for Applied Health Research,University of Birmingham,Birmingham B15 2TT, UK.

120.C.MondinoNational Neurological Institute,Pavia 27100, Italy.

121.Departments of Neurology and Pediatrics, The Johns HopkinsUniversity School of Medicine,Baltimore, MD 21287, USA.

122.Department of Neurology,Admiraal De RuyterHospital, Goes 4462,The Netherlands.

123.Division of Medical Genetics,Department of Pediatrics, Duke University Medical Center, Durham,NC 27710, USA.

124.Department of ComputerScience, New Jersey Institute of Technology,NJ 07102,USA.

125.Department of Pediatric Neurology andDevelopmental Medicine,University Children'sHospital, Tübingen 72076, Germany.

126.Department of Neurosurgery,University of Tübingen, Tübingen72076, Germany.

**STROBE-MR checklist of recommended items to address in reports of Mendelian randomization studies**^1^ ^2^

| **Item No.** | **Section** | **Checklist item** | **Page No.** | **Relevant text from manuscript** |
| --- | --- | --- | --- | --- |
| 1 | **TITLE and ABSTRACT** | Indicate Mendelian randomization (MR) as the study’s design in the title and/or the abstract if that is a main purpose of the study | 2 | The Impact of Serum Magnesium and Calcium on the Risk of Epilepsy: A Mendelian Randomization Study. |
|  | **INTRODUCTION** |  |  |  |
| 2 | **Background** | Explain the scientific background and rationale for the reported study. What is the exposure? Is a potential causal relationship between exposure and outcome plausible? Justify why MR is a helpful method to address the study question | 3-4 |  |
| 3 | **Objectives** | State specific objectives clearly, including pre-specified causal hypotheses (if any). State that MR is a method that, under specific assumptions, intends to estimate causal effects | 4 | Mendelian randomization (MR) is a genetic epidemiologic method by using genetic variants associated with exposures, which can avoid many of the potential methodological limitations of observational studies, such as reverse causation bias and confounding. We conducted this MR study to examine the causal relationship between epilepsy and serum magnesium and calcium concentrations. |
|  | **METHODS** |  |  |  |
| 4 | **Study design and data sources** | Present key elements of the study design early in the article. Consider including a table listing sources of data for all phases of the study. For each data source contributing to the analysis, describe the following: | 5 eTable 1 | Supplementary eTable 1 |
|  | a) | Setting: Describe the study design and the underlying population, if possible. Describe the setting, locations, and relevant dates, including periods of recruitment, exposure, follow-up, and data collection, when available. | 5 |  |
|  | b) | Participants: Give the eligibility criteria, and the sources and methods of selection of participants. Report the sample size, and whether any power or sample size calculations were carried out prior to the main analysis | 5, eTable 1 |  |
|  | c) | Describe measurement, quality control and selection of genetic variants | 4-5 |  |
|  | d) | For each exposure, outcome, and other relevant variables, describe methods of assessment and diagnostic criteria for diseases | 5 |  |
|  | e) | Provide details of ethics committee approval and participant informed consent, if relevant | 10 | This study only used publicly available data. Appropriate ethical approval and participant consent can be acquired in the original publications. |
| 5 | **Assumptions** | Explicitly state the three core IV assumptions for the main analysis (relevance, independence and exclusion restriction) as well assumptions for any additional or sensitivity analysis | 4, Figure 1 | The MR study builds on three predominant assumptions (eFigure 1). |
| 6 | **Statistical methods: main analysis** | Describe statistical methods and statistics used | 5 |  |
|  | a) | Describe how quantitative variables were handled in the analyses (i.e., scale, units, model) | 5 | Odds ratios (ORs) were presented for each 1 SD difference in serum magnesium concentrations (equating to about 0.1 mmol/L) and serum calcium concentrations (equating to about 0.5 mg/dL). |
|  | b) | Describe how genetic variants were handled in the analyses and, if applicable, how their weights were selected | 5 |  |
|  | c) | Describe the MR estimator (e.g. two-stage least squares, Wald ratio) and related statistics. Detail the included covariates and, in case of two-sample MR, whether the same covariate set was used for adjustment in the two samples | Not applicable |  |
|  | d) | Explain how missing data were addressed | Not applicable |  |
|  | e) | If applicable, indicate how multiple testing was addressed | Not applicable |  |
| 7 | **Assessment of assumptions** | Describe any methods or prior knowledge used to assess the assumptions or justify their validity | 5 | In the main analyses, we applied the random-effects inverse-variance weighted (IVW) approach to obtain causal estimates. |
| 8 | **Sensitivity analyses and additional analyses** | Describe any sensitivity analyses or additional analyses performed (e.g. comparison of effect estimates from different approaches, independent replication, bias analytic techniques, validation of instruments, simulations) | 5 | We conducted several sensitivity analyses to identify potential pleiotropy: 1) Cochran's Q test, which was used to evaluate the heterogeneity among different instrumental variables;21 2) weighted median method, which allowed less than 50% of the genetic variants to be invalid instrumental variables;22 3) MR-Egger method, which can detect and adjust pleiotropic bias |
| 9 | **Software and pre-registration** |  | 5 |  |
|  | a) | Name statistical software and package(s), including version and settings used | 5 | All analyses were conducted by using TwoSampleMR and MR-PRESSO packages in R software (Version 4.1.3). |
|  | b) | State whether the study protocol and details were pre-registered (as well as when and where) | No |  |
|  | **RESULTS** |  |  |  |
| 10 | **Descriptive data** |  |  |  |
|  | a) | Report the numbers of individuals at each stage of included studies and reasons for exclusion. Consider use of a flow diagram | eTables 2-4. | eTables 2-4 |
|  | b) | Report summary statistics for phenotypic exposure(s), outcome(s), and other relevant variables (e.g. means, SDs, proportions) | eTables 2-4. | eTables 2-4. |
|  | c) | If the data sources include meta-analyses of previous studies, provide the assessments of heterogeneity across these studies | Not applicable |  |
|  | d) | For two-sample MR:  i.  Provide justification of the similarity of the genetic variant-exposure associations between the exposure and outcome samples  ii.  Provide information on the number of individuals who overlap between the exposure and outcome studies | eTable 2-4.  eTable 1. | eTable 2-4.  eTable 1. |
| 11 | **Main results** |  | 5-7 |  |
|  | a) | Report the associations between genetic variant and exposure, and between genetic variant and outcome, preferably on an interpretable scale | 6 |  |
|  | b) | Report MR estimates of the relationship between exposure and outcome, and the measures of uncertainty from the MR analysis, on an interpretable scale, such as odds ratio or relative risk per SD difference | 6 |  |
|  | c) | If relevant, consider translating estimates of relative risk into absolute risk for a meaningful time period | Not applicable. |  |
|  | d) | Consider plots to visualize results (e.g. forest plot, scatterplot of associations between genetic variants and outcome versus between genetic variants and exposure) | Figure 1,2 | Figure 1 |
| 12 | **Assessment of assumptions** |  |  |  |
|  | a) | Report the assessment of the validity of the assumptions | 6 |  |
|  | b) | Report any additional statistics (e.g., assessments of heterogeneity across genetic variants, such as *I^2^*, Q statistic or E-value) | 6,7 |  |
| 13 | **Sensitivity analyses and additional analyses** |  | 6 |  |
|  | a) | Report any sensitivity analyses to assess the robustness of the main results to violations of the assumptions | 6 |  |
|  | b) | Report results from other sensitivity analyses or additional analyses | 7 | The combined associations of genetic predicted serum magnesium concentrations with overall epilepsy reached at P = 0.002 (OR = 0.28, 95% CI, 0.12 - 0.62) in meta-analyses of data from two consortiums (Figure 2). |
|  | c) | Report any assessment of direction of causal relationship (e.g., bidirectional MR) | Not used |  |
|  | d) | When relevant, report and compare with estimates from non-MR analyses | Not used |  |
|  | e) | Consider additional plots to visualize results (e.g., leave-one-out analyses) | Not used |  |
|  | **DISCUSSION** |  | 7-9 |  |
| 14 | **Key results** | Summarize key results with reference to study objectives | 7 |  |
| 15 | **Limitations** | Discuss limitations of the study, taking into account the validity of the IV assumptions, other sources of potential bias, and imprecision. Discuss both direction and magnitude of any potential bias and any efforts to address them | 8,9 |  |
| 16 | **Interpretation** |  | 9 |  |
|  | a) | Meaning: Give a cautious overall interpretation of results in the context of their limitations and in comparison with other studies | 8,9 | Then, we pooled another large dataset (FinnGen) to further confirm the potential link, which revealed a similar phenomenon. But we supposed that caution should be taken when extrapolating these findings to the clinical situation.  In conclusion, current MR analysis did not support a causal relationship between serum magnesium and epilepsy. In contrast, serum calcium was causally associated with a reduced risk of generalized epilepsy. Further replication of these analyses using even larger GWASs are required. |
|  | b) | Mechanism: Discuss underlying biological mechanisms that could drive a potential causal relationship between the investigated exposure and the outcome, and whether the gene-environment equivalence assumption is reasonable. Use causal language carefully, clarifying that IV estimates may provide causal effects only under certain assumptions | 8 |  |
|  | c) | Clinical relevance: Discuss whether the results have clinical or public policy relevance, and to what extent they inform effect sizes of possible interventions | Not applicable |  |
| 17 | **Generalizability** | Discuss the generalizability of the study results (a) to other populations, (b) across other exposure periods/timings, and (c) across other levels of exposure | 9 | most of participants in this study were of European descent, which limited our findings to extend to other ancestries. |
|  | **OTHER INFORMATION** |  |  |  |
| 18 | **Funding** | Describe sources of funding and the role of funders in the present study and, if applicable, sources of funding for the databases and original study or studies on which the present study is based | 10 | This study was supported by the National Natural Science Foundation of China (81870964). |
| 19 | **Data and data sharing** | Provide the data used to perform all analyses or report where and how the data can be accessed, and reference these sources in the article. Provide the statistical code needed to reproduce the results in the article, or report whether the code is publicly accessible and if so, where | 10 | Genetic variants used can be obtained in the original studies (doi.org/10.1371/journal.pgen.1001045, doi.org/10.1161/CIRCGEN.120.003231). The summary statistics from the GWAS studies for epilepsy are publicly available through the ILEA (www.epigad.org/gwas_ilae2018_16loci.html) and FinnGen (www.finngen.fi). |
| 20 | **Conflicts of Interest** | All authors should declare all potential conflicts of interest | 10 | The authors report no disclosures relevant to the manuscript. |

This checklist is copyrighted by the Equator Network under the Creative Commons Attribution 3.0 Unported (CC BY 3.0) license.

1. Skrivankova VW, Richmond RC, Woolf BAR, Yarmolinsky J, Davies NM, Swanson SA, et al. Strengthening the Reporting of Observational Studies in Epidemiology using Mendelian Randomization (STROBE-MR) Statement. JAMA. 2021;under review.

2. Skrivankova VW, Richmond RC, Woolf BAR, Davies NM, Swanson SA, VanderWeele TJ, et al. Strengthening the Reporting of Observational Studies in Epidemiology using Mendelian Randomisation (STROBE-MR): Explanation and Elaboration. BMJ. 2021;375:n2233.
